# Co_(1–*x*–*y*)_Fe_*x*_Zn_*y*_‑Glycerolate Microspheres as Electrocatalysts for the Oxygen Evolution Reaction

**DOI:** 10.1021/acsaem.5c01604

**Published:** 2025-08-27

**Authors:** Mesaque C. França, Irlan S. Lima, Alireza Ghorbani, Reza Shahbazian-Yassar, Iranaldo S. da Silva, Auro A. Tanaka, Lúcio Angnes, Josué M. Gonçalves, Pedro de Lima-Neto

**Affiliations:** † Institute of Chemistry, University of São Paulo, Av. Prof. Lineu Prestes 748, São Paulo 05508-000, Brazil; ‡ Department of Analytical Chemistry and Physical Chemistry, Science Center, Federal University of Ceara, Fortaleza, Ceara 60440-900, Brazil; § Department of Mechanical & Industrial Engineering, 201649 University of Illinois at Chicago, Chicago, Illinois 60607, United States; ∥ Department of Chemistry, Federal University of Maranhao, Avenida dos Portugueses, 1966, São Luís, Massachusetts 65080-805, Brazil; ⊥ Mackenzie Presbyterian Institute, Mackenzie Institute for Research in Graphene and Nanotechnologies (MackGraphe), São Paulo, São Paulo 01302-907, Brazil

**Keywords:** metal-glycerolate, ternary coordination compound, water-splitting catalysts, oxygen evolution reaction, CoFeZn-glycerolate

## Abstract

Coordination compounds based on transition metals have attracted significant attention for electrocatalyst applications due to their tunable composition and excellent properties as electrode materials. Herein, the design of ternary CoFeZn-glycerolate (CoFeZn-Gly) as an efficient electrocatalyst for oxygen evolution reaction (OER) in an alkaline medium is reported. The combination of Co, Fe, and Zn in the generated microspheres, approaching equimolar conditions, was noted to tend to generate aggregated spheres with an average size of ∼306 nm. The optimized CoFeZn-Gly OER electrocatalyst showed an overpotential of 335 mV (at a current density of 10 mA cm^–2^) and a Tafel slope of 37.2 mV dec^–1^, having the glassy carbon electrode (GCE) as a substrate. Further, the ternary electrocatalyst delivered good stability with a potential retention of 99.22% after 24 h of chronopotentiometry collected at 10 mA cm^–2^, in a 1.0 M KOH electrolyte.

## Graphical abstract

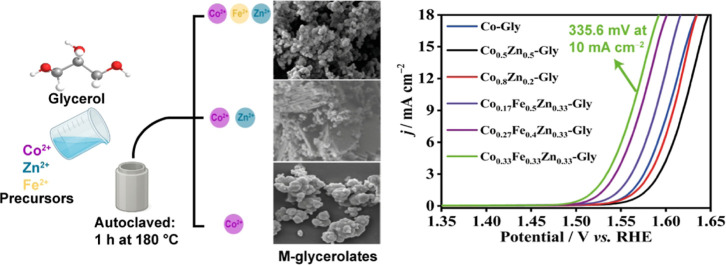

## 1 Introduction

The growing demand for energy worldwide has driven the development of alternative energy storage devices.^1^ Electrochemical water splitting appears as a promising alternative to produce clean energy, since the conversion of water into oxygen (O_2_) and hydrogen (H_2_) has a high potential to compose the energy matrix of the future.^2–4^ However, industrial applications are still hardly connected to the use of precious-based materials such as IrO_2_ and RuO_2_ as anode materials for the use of the OER catalysts. In that context, among the challenges for overcoming this scenario, it is important to highlight the development of efficient, low-cost, and stable electrocatalysts that can accelerate the kinetics of the anodic semireaction of water splitting.

In recent years, various coordination compounds, including metal–organic frameworks (MOFs) and Prussian Blue Analogues (PBAs), have been extensively studied as efficient catalysts for OER, offering opportunities for tuning their composition, structure, and morphology.^5–8^ These possibilities can open a new road to the discovery of coordination-compound-based electrocatalysts that deliver high current density (*j*) and low overpotential (η) under a long period of activity. The recent incorporation of metal glycerolates (M-Glys) into the library of electrocatalysts is a clear indication of the importance of these compounds.^9^ The M-Glys is constituted by a transition metal ion complexed with the ligand (polyalcohol), forming a layered structure, showing remarkable stability, high performance, and well-defined morphology in a wide range of electrochemical applications.^9,10^ In this topic, the use of MOFs and their analogues is known to produce active species of hydroxides and oxyhydroxides (M­(OH)_2_/MO_*x*_(OH)_*y*_) under the OER operations. This transition can generate a low-crystalline or amorphous phase that is usually accompanied by the creation of structural defects and unsaturated metal coordination sites, which can significantly enhance the catalytic effect when compared to pristine material.^11–13^

Therefore, investigations involving Zn-based materials aiming to favor the oxygen evolution reaction (OER) have intensified. Naveen et al.^14^ explored the ZnCo_2_O_4_ properties under OER potentials. As indicated by ex-situ and in situ Raman measurements, the formation of soluble [Zn­(OH)_4_]^2–^ creates defects in the Co_3_O_4_ structure that facilitate the absorption of OER intermediates by the stabilization of the active phase CoO_*x*_(OH)_*y*_ in alkaline conditions.

Considering the properties outlined above, M-Glys has emerged as a promising structural framework with the potential to generate oxyhydroxides functional groups for OER that have already been explored in the literature.^9^ In addition, low conductivity is known to be an intrinsic characteristic of hybrid organic/inorganic materials. The incorporation of additional elements can enhance the electrical conductivity and contribute to the structural evolutions mentioned above.^15,16^ Connecting to that, in this work, the rational design of a ternary CoFeZn-Gly prepared by a solvothermal route with the objective of exploring the potential of Zn in CoFe-Gly for OER catalysis was investigated. The CoFeZn-Gly was extensively characterized in terms of morphological and structural properties as well as electrochemical behavior in a KOH 1.0 M electrolyte. Here, an intense analysis was carried out and a contradiction was found regarding electrochemical active surface area (ECSA), which indicates that our best electrocatalyst with a composition of Co_0.33_Fe_0.33_Zn_0.33_-Gly does not present the highest ECSA, which was attributed to the intrinsic activity of the material, superior to the other compositions made in this work, and will be discussed further.

## 2 Experimental Section

### 2.1 Chemicals

All reagents used were of analytical grade. Iron­(II) acetate (Fe­(CH_3_CO_2_)_2_, 95%) and Nafion (5%) were purchased from Sigma-Aldrich. Cobalt­(II) acetate tetrahydrate (Co­(CH_3_CO_2_)_2·_4H_2_O, 99%), zinc acetate dihydrate (Zn­(CO_2_CH_3_)_2·_2H_2_O, 98%), isopropanol, glycerol, ethanol, and KOH (85%) were purchased from Synth. All aqueous solutions were prepared with ultrapure deionized H_2_O purified with a Milli-Q system.

### 2.2 Materials Synthesis

For the synthesis of Co_1–*x*–*y*_Fe_*x*_Zn_*y*_-Gly compounds, the corresponding metal acetates, always in a total of 2.5 mmol, were mixed in 40 mL of isopropyl alcohol containing 8 mL of glycerol. The ternary glycerolates were prepared using the corresponding metal acetates in the following molar ratios of Co, Fe, and Zn, respectively: 0.17:0.50:0.33 (Co_0.17_Fe_0.50_Zn_0.33_-Gly), 0.27:0.40:0.33 (Co_0.27_Fe_0.40_Zn_0.33_-Gly), and 0.33:0.33:0.33 (Co_0.33_Fe_0.33_Zn_0.33_-Gly). Binary compounds were also synthesized in the following molar ratios of Co and Zn: 0.50:0.50 (Co_0.50_-Zn_0.50_-Gly) and 0.80:0.20 (Co_0.80_-Zn_0.20_-Gly). Lastly, Co-Gly was also synthesized for comparison. In all cases, the resulting solution was stirred for 1 h and then transferred to a stainless steel-lined Teflon autoclave, which was heated in an oven for 1 h at 180 °C. After cooling, the samples were centrifuged, washed twice with ethanol, and dried at 80 °C overnight.

### 2.3 Material Characterization

Scanning electron microscopy (SEM) was performed using a JEOL JSM-IT500HR instrument operating at 5.0 kV with a working distance of 10 mm. Scanning transmission electron microscopy (STEM) analysis was conducted using a JEOL ARM200CF microscope equipped with an aberration-corrected cold field emission source operating at 200 kV and coupled with an Oxford X-max 100TLE windowless X-ray detector. Energy-dispersive X-ray spectroscopy (EDS) mapping was carried out in high-angle annular dark-field (HAADF) mode, where a HAADF detector with a 40 mrad inner detector angle was utilized to collect Z-contrast images at an emission current of 15 μA. For STEM sample preparation, the synthesized coordination polymers were dispersed in ethanol and deposited onto a lacey carbon film supported on a copper grid.

X-ray diffraction (XRD) analysis was performed using a Bruker D2 Phaser diffractometer equipped with a Cu Kα radiation source (λ = 1.5418 Å), operating at 30 kV and 15 mA, with a scanning step size of 0.05° over a 2θ range of 5° to 80°. Fourier-transform infrared (FTIR) spectra were collected using a Bruker ALPHA spectrometer with the samples finely ground and incorporated into KBr pellets.

Surface chemical characterization of the microspheres was conducted using X-ray photoelectron spectroscopy (XPS) with a Specs FlexPS system equipped with an aluminum X-ray source and an energy resolution of 0.1 eV. Spectral calibration was performed by using the C 1s peak at 284.8 eV as a reference. Data analysis was carried out using CasaXPS software (version 2.1).

### 2.4 Electrochemical Characterization

The electrochemical measurements were performed in a potentiostat/galvanostat (Autolab-PGSTAT302N, Metrohm), and all electrochemical tests were carried out in a three-electrode cell. A ring-disc electrode (model 636) equipped with an AFMSRX analytical rotator (Pine Instrument Co.) was associated with the same potentiostat. A glassy carbon electrode (GCE) (area of 0.247 cm^2^) was used as a working electrode, a platinum rod was the counter electrode, and Ag(s)|AgCl(s)|Cl^–^ (aq., 3 mol L^–1^ KCl) was the reference electrode.

The working electrode surface was modified by drop casting of 20 μL of a solution (mass loading = ∼161.6 μg cm^–2^) prepared with 2 mg of catalyst powders and 50 μL of 5% (wt) Nafion dispersed in a 1 mL of a water–ethanol mixture in a ratio of 3:1, which was previously sonicated for 10 min. All electrochemical potentials were converted to the scale of the Reversible Hydrogen Electrode (E_RHE_) according to eq 1 ^17^

1 $$E_{RHE}=E_{Ag/AgCl}+0.059\times pH+0.1976$$

Linear sweep voltammetry (LSV) was recorded without *iR* compensation. The potential was swept between 0.82 and 2.0 V, under a scan rate of 5 mV s^–1^ and at 24 °C. The overpotential (η) at 10 mA cm^–2^ (geometric area) was determined according to eq 2:

2 $$\eta \left(V\right)=E_{RHE}-1.23$$

The Tafel graphs were derived from the LSV curves, and the Tafel slope was calculated by using eq 3.

3 η=a+blog⁡j

In these equations, η, *a*, *b*, *j*, and *j*_0_ refer to the overpotential, the interceptor of the Tafel equation, the Tafel slope, and the applied current density, respectively. Electrochemical Impedance Spectroscopy (EIS) experiments were carried out in the frequency range of 10^5^ to 10^–1^ Hz, at 1.574 V (E vs RHE) and applying 5 mV of AC amplitude.

Cyclic Voltammograms (CVs) were recorded in the non-Faradaic region, cycling the potential between 1.27 and 1.32 V and at different scan rates. These CVs were recorded for the purpose of obtaining the double-layer capacitance (*C*_dl_) in order to estimate the electrochemical surface area (ECSA) of all modified GCEs. The ECSA values were calculated by using eq 4:

4 $$ECSA=R_{f}S$$

In this equation, *S* is the geometric area of the GCE (*S* = 0.247 cm^2^) and *R*_f_ is the roughness factor, which was calculated from eq 5. In these equations, *C*_dl_ is equivalent to the slope of the double-layer charging current versus the slope of the scan rate.

5 $$R_{f}=\frac{C_{dl}}{C_{s}}$$

The specific capacitance (*C*_s_) of the GCE was determined from cyclic voltammetry (CV) measurements carried out in a non-Faradaic region, between 1.27 and 1.32 V (E vs RHE). Prior to the measurements, the electrode was carefully polished until a mirror-like surface was obtained, ensuring reproducibility and cleanliness of the active area. CV curves were recorded at different scan rates (from 5 to 50 mV s^–1^), and the capacitive current was extracted at the midpoint of the potential window. The current was then plotted as a function of the scan rate, resulting in a linear relationship whose slope corresponds to the double-layer capacitance (*C*_dl_). Considering the geometric area of the glassy carbon electrode (*S* = 0.247 cm^2^), the specific capacitance (*C*_s_ = 23.2 μF cm^–2^) was calculated by using eq 6.

6 $$C_{s}=\frac{C_{dl}}{S}$$

In addition, the activation of the modified electrode surface was performed with 20 CV scans from the range of 0.82 to 1.62 V (E vs RHE), and the stability of the materials under OER conditions was investigated with chronopotentiometry experiments carried out for 24 h at a current density of 10 mA cm^–2^. Finally, all electrochemical measurements (on the GCE) were carried out using the rotating disk electrode at 1600 rpm.

## 3 Results and Discussion

### 3.1 Physical Characterization

The X-ray diffractograms and vibrational spectra of the synthesized M-Gly materials are displayed in Figure 1. It can be noted in Figure 1a that Co-Gly, Co_0.8_Zn_0.2_-Gly, and Co_0.33_Fe_0.33_Zn_0.33_-Gly samples have a similar structure, consistent with the presence of a low-angle diffraction peak at 10.7° (0.84 nm), related to the gap between the layers, and another diffraction peak located at 36.0°, both of which consist of stacked metal–oxygen layers separated by bound glycerolate anions, in agreement with the findings of Nguyen et al.^10^ and Wang et al.^18^ and with our previous results.^19,20^ The FTIR spectra, shown in Figure 1b, revealed some important characteristic bands of organic ligands. The signal at 3425 cm^–1^ can be assigned to stretching of the hydroxyl group (O–H). The bands located between 2918 cm^–1^ and 2842 cm^–1^ are correlated to the occurrence of the stretching vibration mode of the C–H bond. The peaks observed at 1576 and 1423 cm^–1^ are characteristic vibrational movements of the O–C–O chemical bond, which is understandable, since glycerol is also oxidized to secondary species, such as the one containing carboxylate groups. Peaks associated with the vibration of the C–O bond are displayed between 1114 and 1004 cm^–1^_,_ while the band centered at 795 cm^–1^ is correlated to the out-of-plane bending vibrations of the C–H bond. The crucial IR band related to metal–oxygen *v*(M–O) is located at 584 cm^–1^, which confirms the presence of a metal–glycerolate bond. Finally, X-ray diffractograms and FTIR spectra indicate that *M*-glycerolates were successfully synthesized.

**Figure 1:**
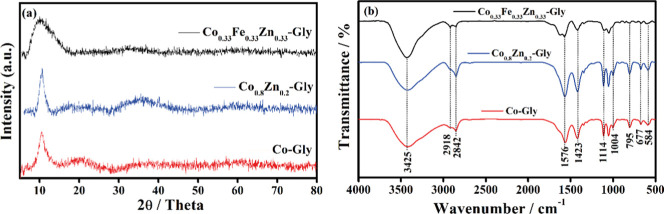
(a) X-ray diffraction and (b) FTIR spectra obtained for the Co-Gly, Co_0.8_Zn_0.2_-Gly, and Co_0.33_Fe_0.33_Zn_0.33_-Gly microspheres.

The SEM images of the Co_(1–*x*–*y*)_Fe_*x*_Zn_*y*_-Gly compounds are shown in Figure 2. It is possible to observe that the synthesized materials exhibited spherical morphology and the formation of clusters, except for the Co_0.5_Zn_0.5_-Gly, which exhibits spherical particles and rod clusters, which is attributed to the higher Zn content in this compound, since the Zn-Gly also exhibits rod assemblies, as shown in Figure S1. The analysis of the histograms, shown in Figure S2a–c, indicated that the Co_0.33_Fe_0.33_Zn_0.33_-Gly had the smallest average particle size (≈306 nm) with a size distribution between 100 and 1000 nm, followed by Co_0.8_Zn_0.2_-Gly and Co-Gly (≈558 nm), with size distributions between 100 and 1400 nm and 100 and 1600 nm, respectively.

**Figure 2:**
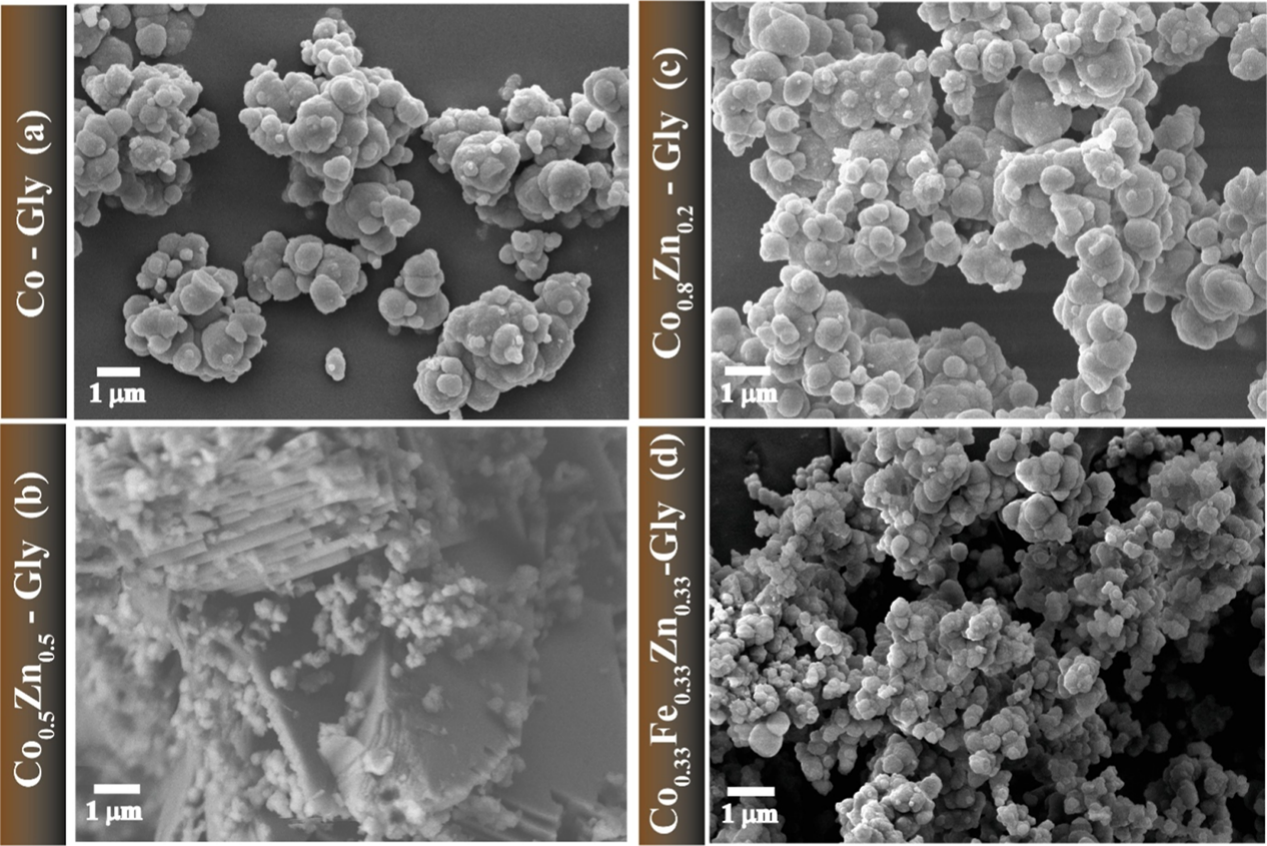
SEM images showing the particles of the synthesized compounds from unary to ternary: (a) Co-Gly, (b) Co_0.5_Zn_0.5_-Gly, (c) Co_0.8_Zn_0.2_-Gly, and (d) Co_0.33_Fe_0.33_Zn_0.33_-Gly.

The elemental distribution, determined by EDS-STEM mapping, showed the distribution of metal ions in the metal-glycerolate particles, and the distributions of elements C and O are shown in Figure 3. As illustrated in Figure 3a, the elemental distribution mapping for Co-Gly and the EDS-STEM layered image shows a uniform distribution of the elements C, O, and Co. It is also evident that both Co_0.8_Zn_0.2_-Gly (Figure 3b) and Co_0.33_Fe_0.33_Zn_0.33_-Gly (Figure 3c) show layered images for the distribution of the C and the O elements and for the Co, Fe, and Zn ions, indicating that the distribution was uniform throughout the binary and ternary coordination polymers. These results corroborate with other studies, showing the efficiency of the proposed synthesis route (solvothermal) in producing multielement materials, guaranteeing a good distribution of the elements in the matrix.

**Figure 3:**
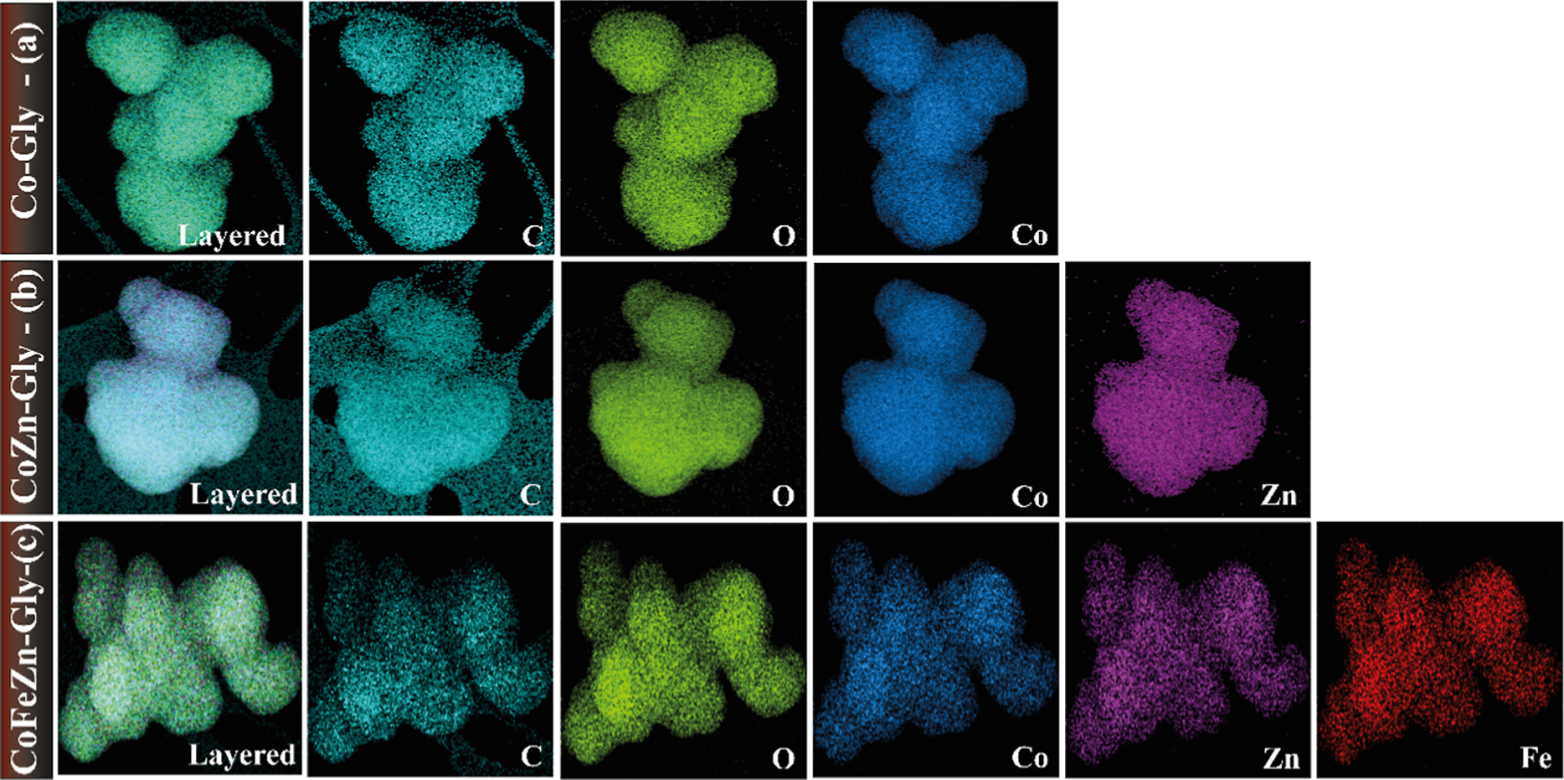
EDS-STEM elemental mapping images of respective (a) unary Co-Gly, (b) binary CoZn-Gly, and (c) ternary CoFeZn-Gly microparticles, all highlighting their spherical morphology. The layered images and the elemental mappings show a uniform mixing of the metal and ligand components.

The valences of the metals were analyzed by X-ray photoelectron spectroscopy (XPS) for the material that exhibited the best electrocatalytic performance, in this case, Co_0.33_Fe_0.33_Zn_0.33_-Gly. Figure 4 shows the high-resolution XPS spectrum for the Co 2p, Fe 2p, and Zn 2p species of the CoFeZn-Gly.

**Figure 4:**
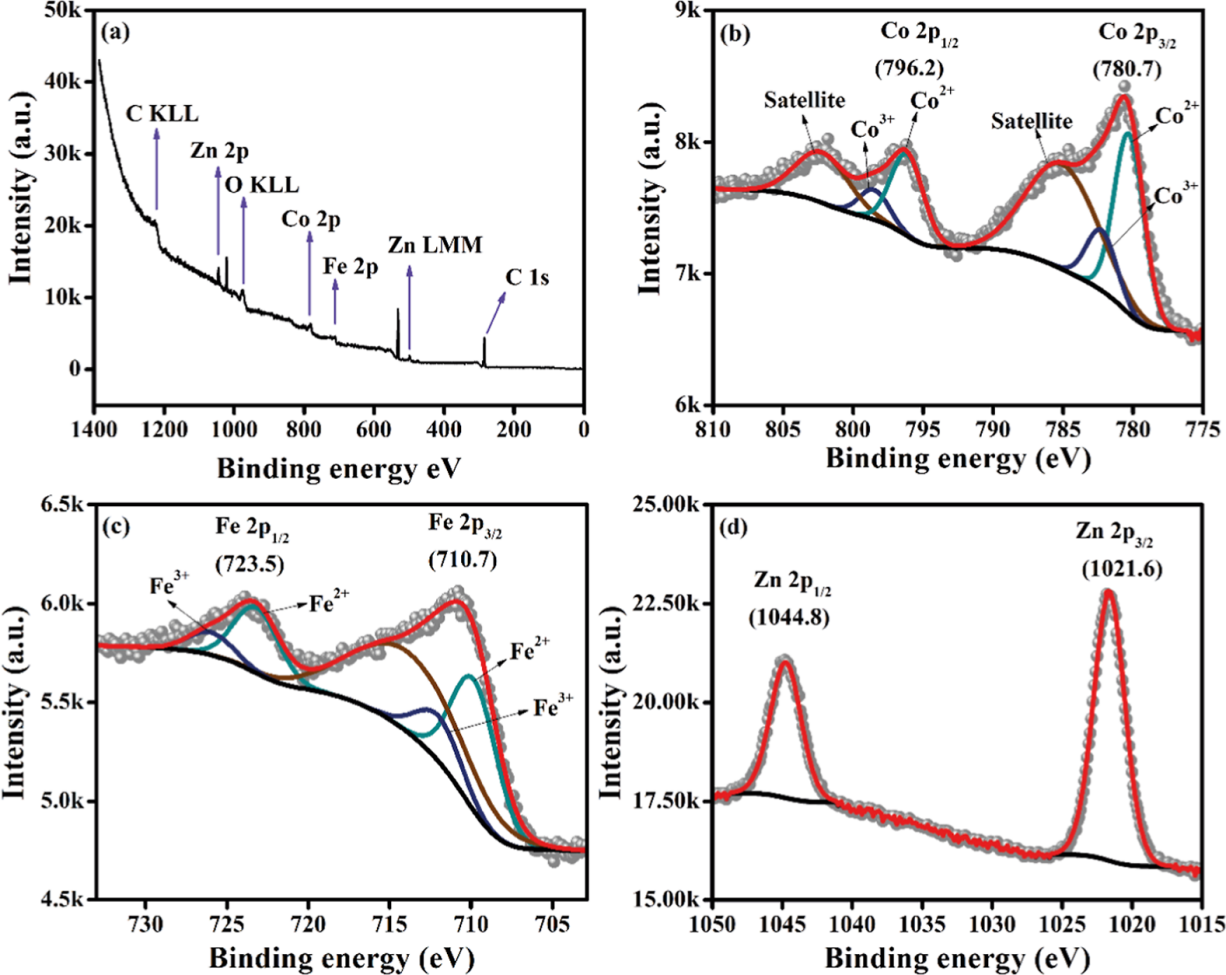
X-ray photoelectron spectroscopy (XPS) of CoFeZn-Gly. (a) Survey spectrum. In the 2p regions of: (b) cobalt, (c) iron, and (d) zinc.

The Co 2p, Fe 2p, and Zn 2p peaks can be fitted into two spin orbits, revealing two valence states for Co (Co^2+^ and Co^3+^) and Fe (Fe^2+^ and Fe^3+^) and one valence state for Zn (Zn^2+^). Figure 4a shows the XPS survey spectra of the CoFeZn-Gly electrocatalyst, where only the elements O, Co, Fe, Zn, and C could be seen, showing that the method used to synthesize the material produces the projected product. Figure 4b shows that the Co^2+^ peaks are in Co 2p3/2 at 854.6 eV and Co 2p1/2 at 871.9 eV, respectively, while the Co^3+^ peaks are seen at 856.0 eV (Co 2p3/2) and 873.9 eV (Co 2p1/2), respectively. Two more satellite peaks are also seen in the Co 2p spectrum at 785.5 and 802 eV.^21^ Like Co 2p, the Fe 2p spectrum also shows the oxidation of some of the Fe^2+^ ions.

In fact, the Fe 2p spectrum shows Fe^2+^ (Fe 2p3/2) and Fe^3+^ (Fe 2p1/2) at 709.8 and 712.0 eV, respectively, and Fe^2+^ (Fe 2p1/2) and Fe^3+^ (Fe 2p3/2) at 726.0 and 723.2 eV, respectively (Figure 4c).^22^ Figure 4d shows the spectrum for Zn 2p (2p3/2 and 2p1/2), which exhibits a single valence at 1021.6 and 1044.8 eV.^23^ The high-resolution C 1s spectrum (Figure S3a) reveals two peaks related to the partial oxidation of glycerol after the solvothermal reaction. The peaks are located, respectively, at 284.7 eV, referring to C–C, and that at 288.5 eV is associated with O–C=O.^24^ The O 1s spectrum (Figure S3b) presents peaks related to four components at 530.4, 531.1, 532.3, and 533 eV, attributed to lattice oxygen O–M, O–H, C–O bonding, and physically absorbed water, respectively.^10^

### 3.2 Determination of the Electrochemical Surface Area (ECSA)

To elucidate the intrinsic activity of the Co_(1–*x*–*y*)_Fe_*x*_Zn_*y*_-Gly samples, the electrochemical double-layer capacitance (*C*_dl_) was obtained by CV, which was calculated from the linear relation of the capacitive current against the scan rate, varied from 5 up to 50 mV s^–1^, and applying a potential range between 1.274 and 1.324 V (E vs RHE), which was chosen surrounding the OCP region, for all specimens. The obtained cyclic voltammograms are displayed in Figure S4.

Figure 5 shows good linear correlations between the differences in anodic (*I*_a_) and cathodic (*I*_c_) capacitive currents against the scan rate. The *C*_dl_ data correspond to the slope of the straight line, and the following values were achieved from linear regression analysis: 2.19 mF for Co-Gly, 2.12 mF for Co_0.8_Zn_0.2_-Gly, 1.53 mF for Co_0.5_Zn_0.5_-Gly, 0.2 mF for Co_0.17_Zn_0.33_Fe_0.5_-Gly, 0.54 mF for Co_0.27_Zn_0.33_Fe_0.4_-Gly, and 0.55 mF for Co_0.33_Zn_0.33_Fe_0.33_-Gly. From these data, the corresponding ECSA values were calculated, resulting in the following values: 22.80 cm^2^ for Co-Gly, 15.93 cm^2^ for Co_0.8_Zn_0.2_-Gly, 22.08 cm^2^ for Co_0.5_Zn_0.5_-Gly, 2.08 cm^2^ for Co_0.17_Zn_0.33_Fe_0.5_-Gly, 5.62 cm^2^ for Co_0.27_Zn_0.33_Fe_0.4_-Gly, and 5.73 cm^2^ for Co_0.33_Zn_0.33_Fe_0.33_-Gly. From these records, it can be noted that ECSA values are related to the Co content in the synthesized compound and that the GCE modified with the ternary compounds exhibits the smallest ECSA.

**Figure 5:**
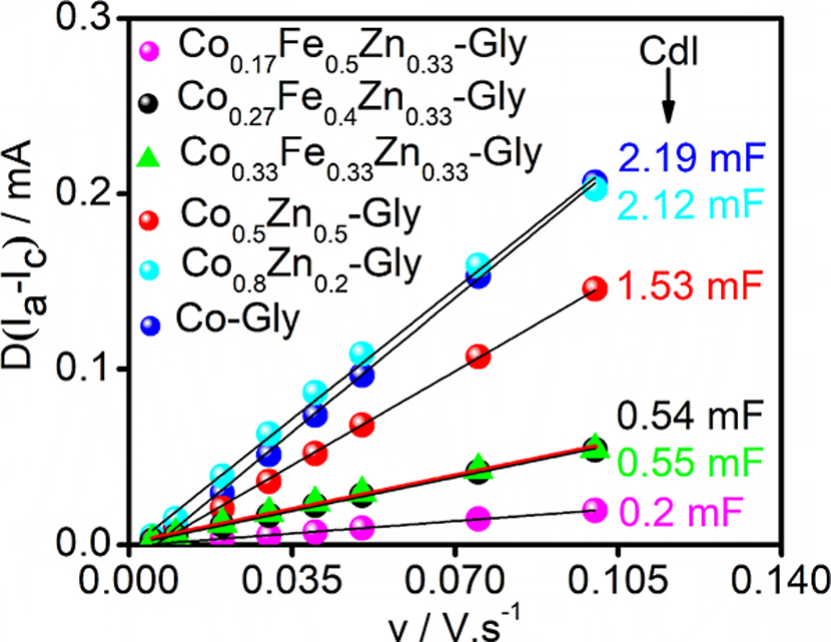
Plot of current density vs scan rates.

### 3.3 OER Performance

To better understand the electrochemical properties of the prepared M-Gly, a series of electrochemical experiments in a 1 mol L^–1^ KOH solution were performed by using a standard three-electrode system mentioned before. Figure 6a displays LSV curves of various CoZnFe-Gly, CoZn-Gly, and the Co-Gly synthesized (Figure S5), where the peak observed is situated close to 1.1 V (E vs RHE), indicating the transition oxidation state of Co^2+^/Co^3+^.^25,26^ From a practical standpoint, in industrial applications, energy consumption is a crucial parameter to consider in the oxygen evolution reaction (OER). In this context, the overpotential required to achieve a current density of 10 mA cm^–2^ (geometric area) is the most commonly used parameter to classify the performance of electrocatalysts. However, in this situation, electrocatalytic effects are mixed with surface roughness effects, meaning that the results do not accurately represent the intrinsic electrocatalytic activity of the material. To overcome this issue, the LSV curves used for the Tafel plot (Figure 6c) were normalized by the corresponding electrochemically active surface area (ECSA, Table S1).

**Figure 6:**
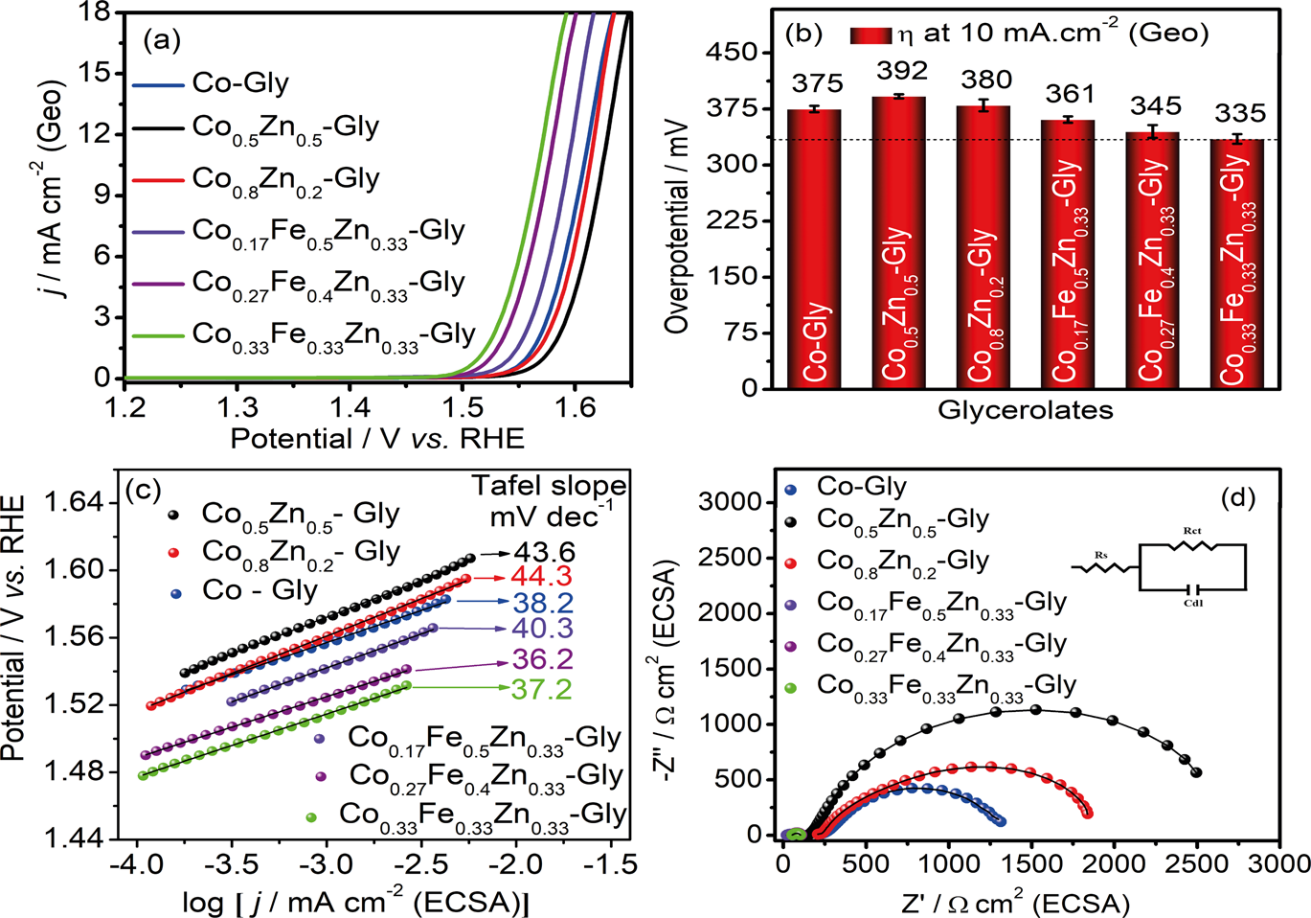
(a) LSV curves (on an RDE at 1600 rpm in a 1.0 mol L^–1^ KOH solution, scan rate: 5 mV s^–1^), (b) overpotential at a current density of 10 mA cm^–2^, (c) Tafel plots, and (d) Nyquist plots normalized by ECSA for Co-Gly, Co_0.5_Zn_0.5_-Gly, Co_0.8_Zn_0.2_-Gly, Co_0.17_Fe_0.5_Zn_0.33_-Gly, Co_0.27_Fe_0.4_Zn_0.33_-Gly, and Co_0.33_Fe_0.33_Zn_0.33_-Gly. Error bars represent standard deviations based on triplicate measurements.

For an electrocatalyst performance comparison, the overpotential was calculated at a current density of 10 mA cm^–2^ (η_10_). Initially, the binary CoZn-Gly specimens exhibited the poorest electroactivity, as revealed by the η_10_ of 392 and 380 mV for Co_0.5_Zn_0.5_-Gly and Co_0.8_Zn_0.2_-Gly, respectively. The Co-Gly performance (η_10_ = 375 mV) was overcome only with the addition of iron in the CoZn-Gly structure, as shown in Figure 6b. Three distinct proportions of these metal precursors presented interesting OER catalytic activity, where the ternary configuration of Co_0.33_Fe_0.33_Zn_0.33_-Gly presented the lowest overpotential (η_10_ = 335 mV), followed by Co_0.27_Fe_0.4_Zn_0.33_-Gly (η_10_ = 345 mV), and Co_0.17_Fe_0.5_Zn_0.33_-Gly with an overpotential of 361 mV.

To gain more insight into the kinetics of OER, the Tafel plot of all specimens was also analyzed across the linear region of the overpotential vs log (*j*), as shown in Figure 6c. The Co_0.33_Fe_0.33_Zn_0.33_-Gly, Co_0.27_Fe_0.4_Zn_0.33_-Gly, Co_0.17_Fe_0.5_Zn_0.33_-Gly, Co-Gly, Co_0.8_Zn_0.2_-Gly, and Co_0.5_Zn_0.5_-Gly presented a Tafel slope of 37.2 ± 2, 36.2 ± 3, 40.3 ± 1, 38.2 ± 2, 44.3.6 ± 3, and 43.6 ± 1 mV dec^–1^, respectively. As expected, the presence of Co ions is a crucial feature for a high OER electrocatalytic kinetic rate, according to the low Tafel slope of CoZnFe-Gly in equimolar proportions, as well as other ternary compositions.

To elucidate the synergism between these metals, electrochemical impedance spectroscopy (EIS) of the above samples was measured at a potential of 1.57 V (vs RHE), and all curves were fitted according to the Randles circuit (Figure 6). It is noted that Co_0.33_Fe_0.33_Zn_0.33_-Gly exhibited the lowest transfer resistance (*R*_ct_ of 50.65 Ω·cm^2^) in the OER conditions, followed by their analogues Co_0.27_Fe_0.4_Zn_0.33_-Gly and Co_0.17_Fe_0.5_Zn_0.33_-Gly with an *R*_ct_ of 57.9 Ω·cm^2^ and 39.8 Ω·cm^2^, respectively. The Co_0.5_Zn_0.5_-Gly possessed the highest semicircle with an *R*_ct_ of 2.535 k Ω·cm^2^, thus the lowest rate of electron transfer during the OER mechanism. All values are seen in Table 1, as well as the values achieved for the solution resistance (*R*_s_).

**Table 1:** Charge Transfer Resistance (*R*_ct_) Values for a Parallel RC Circuit

M-Gly	C/μF	*R*_ct_/Ω·cm^2^	fitting
Co	74.6	1.1 k	2.17 × 10^–3^
Co_0.5_Zn_0.5_	31.5	2.535 k	3.99 × 10^–3^
Co_0.8_Zn_0.2_	61.8	1.294 k	2.17 × 10^–3^
Co_0.17_Fe_0.5_Zn_0.33_	200	39.8	7.90 × 10^–3^
Co_0.27_Fe_0.4_Zn_0.33_	43.6	57.9	1.05 × 10^–2^
Co_0.33_Fe_0.33_Zn_0.33_	99.4	50.65	1.95 × 10^–3^

The formation of CoOOH is a determining factor for the activity of an electrocatalyst in OER. This process can be enhanced with the addition of Fe^3+^, which acts as a modulating agent on the electronic structure through the formation of Fe-CoOOH.^27,28^ Furthermore, the presence of zinc and its characteristic leaching effect in an alkaline medium can create oxygen vacancies on the catalyst surface, thereby increasing the affinity for the absorbed intermediates in the OER activity.

The stability test showed excellent stability for 24 h of continuous measurements, with a potential retention of 99.2%, as shown in Figure 7. It becomes evident that the intrinsic activity of the active sites for Co_0.33_Fe_0.33_Zn_0.33_-Gly (Figure S6) is the predominant factor for the observed electrocatalytic performance. This is justified because the structure of M-Glys favors the modulation effect of electronic properties caused by the variation in the equimolar metal composition of the catalyst, promoting the formation of new active sites.^29^ In the work of Huang et al.,^1^ the synthesis of two-dimensional CoZnFe-LDH nanosheets via a coprecipitation method was reported. The produced Co_0.7_ZnFe_0.3_-LDH revealed an η of 350 mV and a Tafel slope of 63 mV dec^–1^ at a current density of 10 mA cm^–2^ and exhibited 99% potential retention over a stability of 10 h in 1 M KOH. This indicates that the proposed catalyst is efficient for the OER. Fe doping improved the carrier density and can also activate the coactive sites, thus lowering the energy barrier for the transformation of OH* to OOH*, leading to a significant improvement in the OER performance. In another example, the formation of double hydroxide layers in Ni/Co oxyhydroxide is favored, with more accessible active centers, due to the presence of Fe^3+^ ions.^30,31^

**Figure 7:**
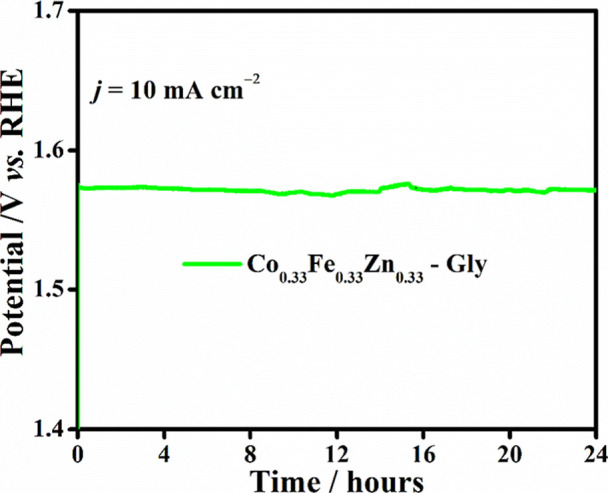
OER stability test at 10 mA cm^–2^.

Furthermore, based on those findings, the ternary glycerolate catalyst proposed here can present the well-known OER mechanism. It starts with the adsorption of OH^–^ ions on the active metal centers (M*) to generate metal hydroxyls (M–OH) with the release of one electron. In the sequence, the M–OH absorbs another OH^–^ ion and releases electrons; such a transition converts into the active intermediate hydroperoxide (M–OOH). The final steps occur through the proton-coupled electron transfer with the release of O_2_ and the back at the initial step by the regeneration of active site M*,^32^ as highlighted in the following equations:

7 $${Μ}^{*}+Ο{Η}^{-}\to ΜΟΗ+e^{-}$$

8 $$ΜΟΗ+Ο{Η}^{-}\to ΜΟ+{Η}_{2}Ο+e^{-}$$

9 $$ΜΟ+Ο{Η}^{-}\to ΜΟΟΗ+e^{-}$$

10 $$ΜΟΟΗ+Ο{Η}^{-}\to {Η}_{2}Ο+Μ{Ο}_{2}+e^{-}$$

11 $$Μ{Ο}_{2}\to M^{*}+O_{2}$$

As a comparison, Zhou et al.^33^ proposed an activation method onto a commercial NiFe foam (NFF) by CV, which consists of a spontaneous corrosion combined with the addition of Zn­(NO_3_)_2_ and Ni­(NO_3_)_2_ solution. The fabricated NiFeZn-LDH/NFF showed an interesting h of 565 mV at a high current density of 400 mA cm^–2^, in conditions of 0.1 M of Ni­(NO_3_)_2_ and 0.04 M of Zn­(NO_3_)_2_ precursor concentrations. As pointed out by in situ Raman spectroscopy, the NiFeOOH formed during OER catalysis is the active phase, while the dissolution of Zn^2+^ in cationic vacancies offered superior OER stability of up to 112 h in 1 M KOH. Similar behavior is found in the work of Zhang et al.,^34^ where the synthesis of a ternary catalyst composed of CoFeZn nanoparticles supported on N-doped carbon (CoFeZn/NC) was proposed using a one-step pyrolysis method. In this case, ZIF-8 (Zn), ZIF-67 (Co), and MUV-3 (Fe) were decomposed into N-doped porous carbon (NC) and metallic nanoparticles, resulting in a CoFeZn/NC composite. Furthermore, Fe and Co were found in the form of metallic nanoparticles with a small amount of Zn particles, as pointed out by the XRD measurements. Additionally, the partial volatilization of Zn species caused vacancy exposure, which enabled the formation of more abundant Fe and Co active sites. Thus, the proposed CoFeZn/NC exhibited an excellent η of 319.9 mV at a current density of 10 mA cm^–2^ and stability of up to 5.55 h, in 1 M KOH. The results of the electrocatalytic activity for OER in the present study can be easily compared with other studies already published, as shown in Table 2. In fact, the results of the CoFeZn-Gly based catalysts present good similarity with the data already reported in the literature. Therefore, many of these catalysts presented in Table 2 require more than one step of synthesis, thus consuming more reagents and time, while the method proposed here involves the use of metal salts, glycerol, and isopropanol that generate the catalyst in a 2 h reaction and can be scaled up by proportionally increasing the amount of reactants.

**Table 2:** Comparison of the Catalytic Performance of the OER of the Co_0.27_Fe_0.4_Zn_0.5_- and Co_0.33_Fe_0.33_Zn_0.33_-Glycerolate with Other Reported Works

electrocatalyst	synthesis method	η_10_ (10 mA cm^–2^)	Tafel slope (mV dec^–1^)	stability (hrs)	ref
CoFe LDH	coprecipitation	400	83.0		^1^
CoFeZn/NC	pyrolysis	319	82.4	5.55	^34^
NiFeZn-LDH/NFF	spontaneous corrosion and CV activation	565 at 400 mA cm^–2^	38.0	112	^33^
Co–Ni–Fe-LDHs	chemical precipitation	322	41.8	48	^35^
NiCo-*t*-MOF/CC		440 at 100 mA cm^–2^	83.0	62	^13^
CoOBrPc + KB		381	64.0	80	^36^
Co_0.27_Fe_0.4_Zn_0.33_-glycerolate	solvothermal	345	36.2	24	this work
Co_0.33_Fe_0.33_Zn_0.33_-glycerolate	solvothermal	335	37.2	24	this work

## 4 Conclusion

In summary, the metal-glycerolates (Co-Gly, CoZn-Gly, and CoFeZn-Gly) synthesized using the solvothermal method showed high levels of OER activity and can be used in alkaline electrolytes. These findings suggest that these metal-glycerolates have significant potential as catalysts for energy conversion processes. Further studies will be essential to exploring their stability and performance in practical applications. Among the electrocatalysts, Co_0.33_Fe0_.33_Zn_0.33_-Gly showed the best activity and requires an η of 335.6 mV to reach *j* 10 mA cm^–2^, with a low Tafel slope of 36.9 mV dec^–1^, as well as showing excellent stability for 24 h in alkaline media. The superior performance of the OER is attributed to the Co^3+^ and Fe^3+^ ions, which are able to stabilize the high valence active sites. An interesting fact was observed: In the study of the area, the highest ECSA was for Co-Gly, followed by those for Co_0.8_Zn_0.2_-Gly and Co_0.5_Zn_0.5_-Gly. These results indicate that the increase in the area is related to the Co concentration. However, the best performance was achieved by Co_0.33_Fe_0.33_Zn_0.33_-Gly. The data measured for these materials suggest that the Co_0.33_Fe_0.33_Zn_0.33_-Gly has different exposure sites, which in this case are more active sites, responsible for the improvement in its activity. This occurred due to an effect known as the intrinsic activity of the material, which is defined by the equation: Intrinsic Activity = *j*/ECSA, where *j* is the current density. Therefore, a material with a lower ECSA may still possess more active catalytic sites, even if they are smaller in number. This means that each active site individually exhibits a higher efficiency in catalyzing the reaction, which compensates for the lower total number of sites. This work shows a cheap catalyst with superior catalytic activity, thus opening the door to its practical energy storage in a wide range of electrocatalytic fields.

## Supplementary Material



## Notes

SEM images, distribution of particles, C 1s and O 1s high-resolution spectra, double-layer capacitance measurements, LSV curves, intrinsic activity, and electrochemical surface area (PDF)
