# Supplementary material for: Co(1–x–y)Fe x Zn y ‑Glycerolate Microspheres as Electrocatalysts for the Oxygen Evolution Reaction
Source: ACS Appl Energy Mater. 2025 Aug 27;8(17):12618–26. doi: 10.1021/acsaem.5c01604 (PMC12421500; doi:10.1021/acsaem.5c01604)
Supplement: Supplementary file 1 [file ae5c01604_si_001.pdf]

Supporting Information

$Co_{(1-x-y)}Fe_xZn_y$-Glycerolate microspheres as electrocatalysts for the oxygen evolution reaction

Mesaque C. França[a,b], Irlan S. Lima[a], Alireza Ghorbani[c], Reza Shahbazian-Yassar[c], Iranaldo S. da Silva [d], Auro A. Tanaka[d], Lúcio Angnes[a,]*, Josué M. Gonçalves [e,]*, Pedro de Lima-Neto [b,] *

[a] Institute of Chemistry, University of São Paulo, Av. Prof. Lineu Prestes 748, São Paulo, 05508-000, Brazil

[b] Department of Analytical Chemistry and Physical Chemistry, Science Center, Federal University of Ceara, Fortaleza, CE, 60440-900, Brazil

[c] Department of Mechanical & Industrial Engineering, University of Illinois at Chicago, Chicago, Illinois 60607, United States

[d] Department of Chemistry, Federal University of Maranhão, Avenida dos Portugueses, 1966, São Luís, Maranhão 65080-805, Brazil

[e] Mackenzie Institute for Research in Graphene and Nanotechnologies (MackGraphe), Mackenzie Presbyterian Institute, São Paulo, 01302-907, Brazil

Corresponding authors:

E-mail address: pln@ufc.br *(Pedro de Lima-Neto),* luangnes@iq.usp.br *(Lucio Angnes) and* josuefisicoquimico@hotmail.com *(Josué M. Gonçalves)*

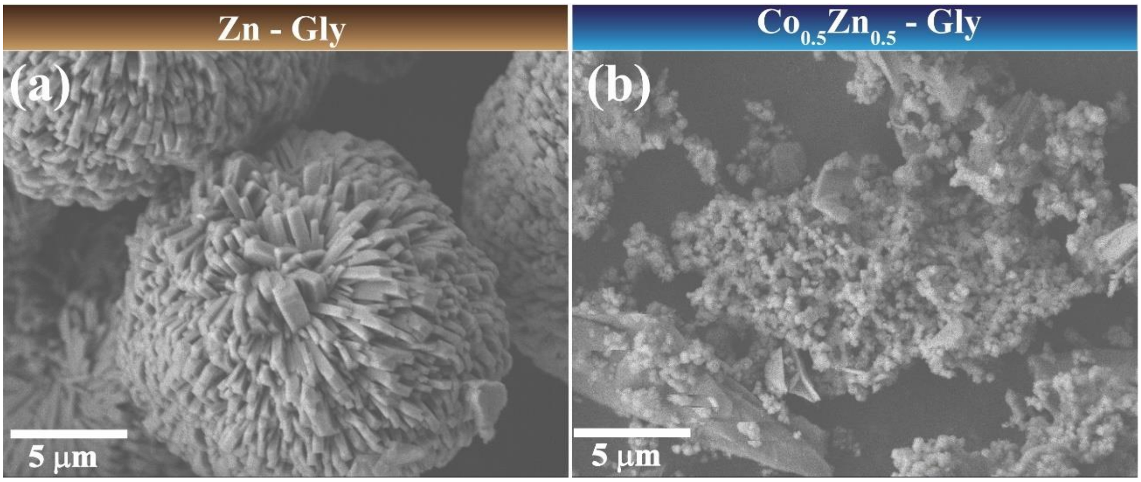

Figure S1. SEM images showing (a) Zn-Gly and (b) $Co_{0.5}Zn_{0.5}$-Gly. The magnification is × 5.000.

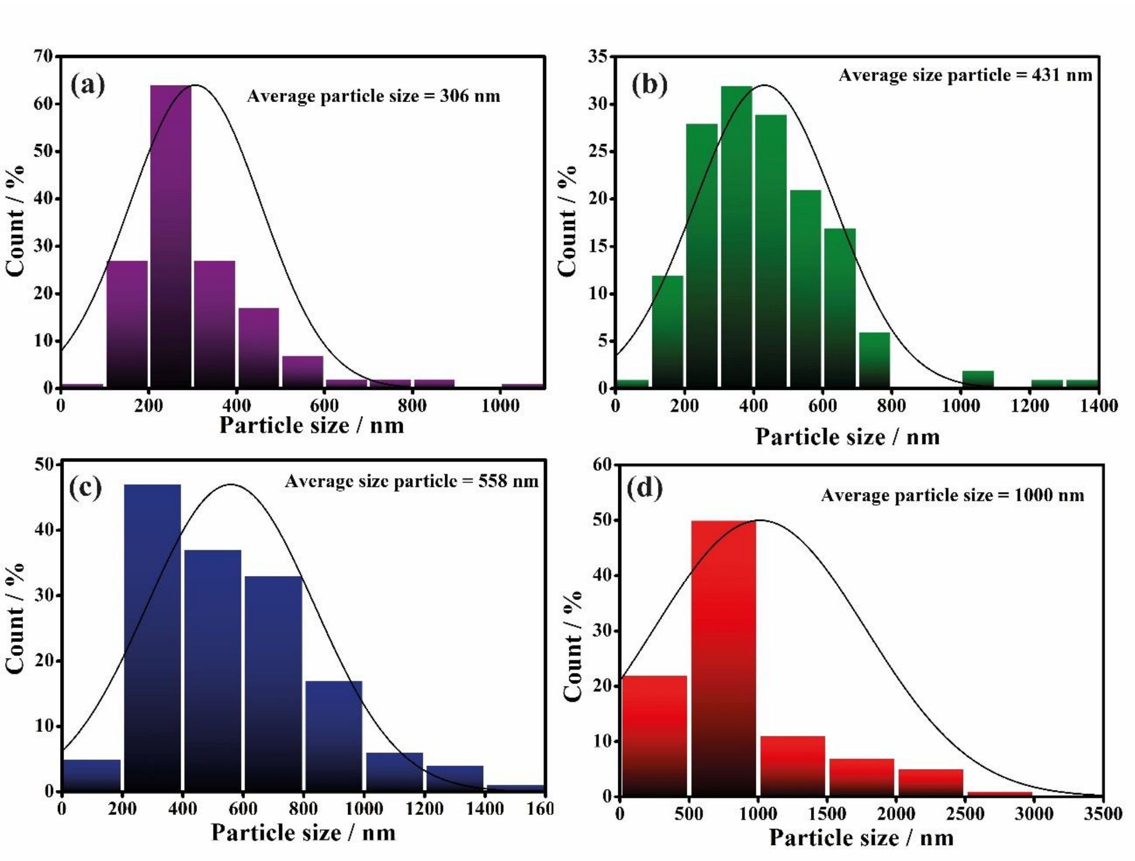

Figure S2. Distribution of the particle for (a) $Co_{0.33}Fe_{0.33}Zn_{0.33}$-Gly, (b) $Co_{0.8}Zn_{0.2}$-Gly, (c) Co-Gly, and Co-Zn-Gly.

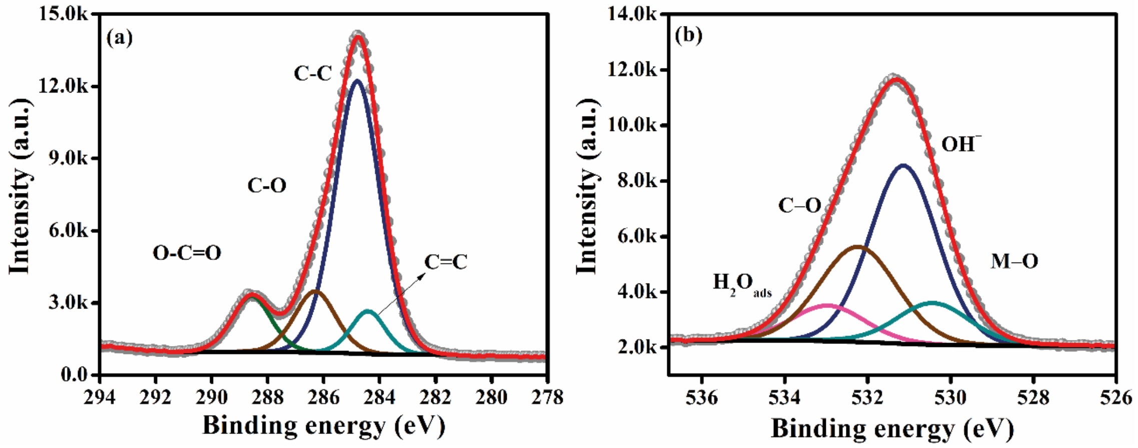

Figure S3. (a) C1s and (b) O1s high-resolution spectra for the CoFeZn-Gly electrode material.

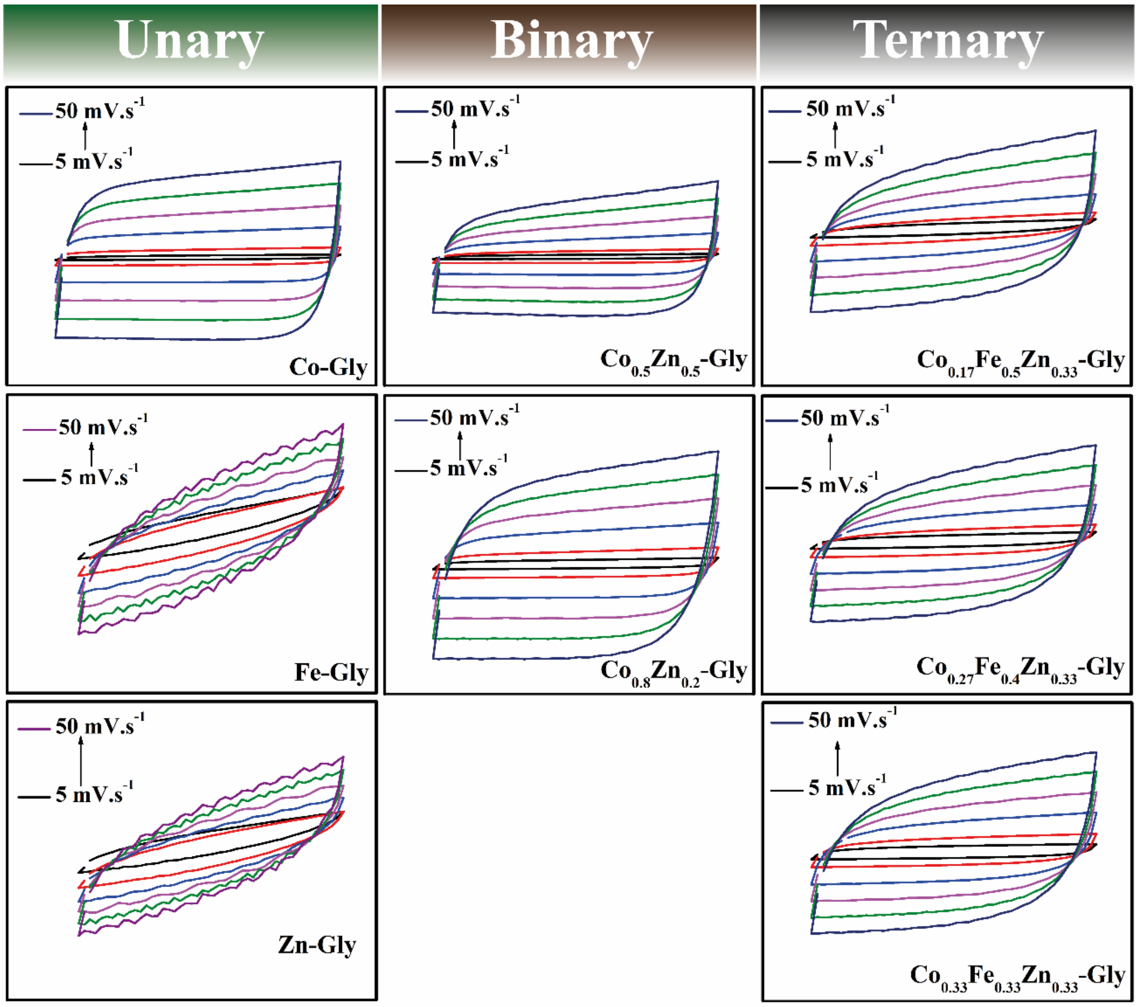

Figure S4. Double-layer capacitance measurements for determining electrochemically active surface area from cyclic voltammetry measurements as a function of scan rate: 0.005, 0.01, 0.02, 0.03, 0.04, and V s^{-1}, for unary (Zn-Gly, Fe-Gly, Co-Gly), binary ($Co_{0.5}Zn_{0.5}$-Gly, $Co_{0.8}Zn_{0.2}$-Gly), and ternary M-Gly ($Co_{0.17}Fe_{0.5}Zn_{0.33}$ -Gly, $Co_{0.27}Fe_{0.4}Zn_{0.33}$-Gly, and $Co_{0.33}Fe_{0.33}Zn_{0.33}$-Gly) on an RDE at 1600 rpm in a 1.0 mol L^{-1} KOH solution.

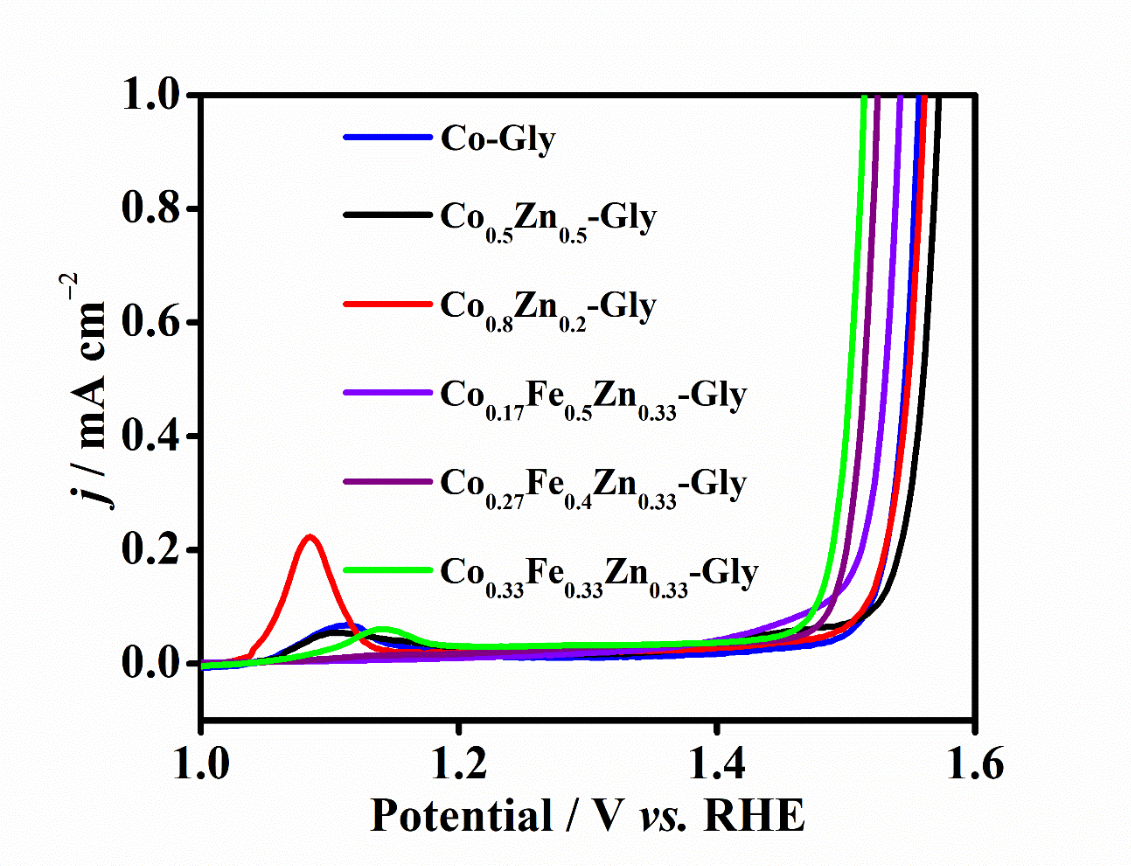

Figure S5. The LSV curves of the different synthesized materials: CoZnFe-Gly, CoZn-Gly, and Co-Gly, indicate the Co^{2+}/Co^{3+} oxidation transition near 1.1 V (E *vs*. RHE), in 1 M KOH at a scan rate of 5 mV s^{-1}.

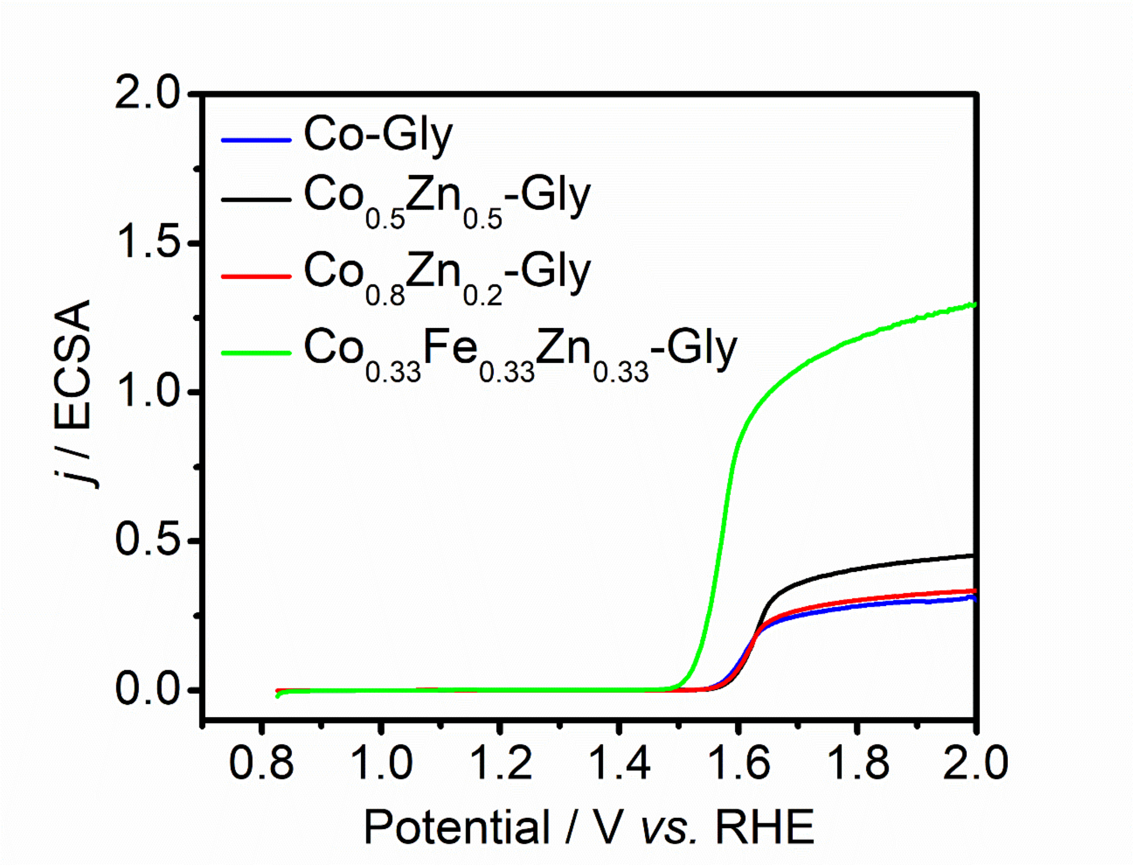

Figure S6. Intrinsic activity for Co-Gly, $Co_{0.5}Zn_{0.5}$-Gly, $Co_{0.8}Zn_{0.2}$-Gly, and $Co_{0.33}Fe_{0.33}Zn_{0.33}$-Gly.

Table S1. Electrochemical Surface Area (ECSA).

Glycerolates	Rf	ECSA / cm^2
**Co-Gly**	92.3	22.80
**$Co_{0.5}Zn_{0.5}$-Gly**	64.5	15.93
**$Co_{0.8}Zn_{0.2}$-Gly**	89.4	22.08
**$Co_{0.17}Fe_{0.5}Zn_{0.33}$-Gly**	8.4	2.08
**$Co_{0.27}Fe_{0.4}Zn_{0.33}$-Gly**	22.8	5.62
**$Co_{0.33}Fe_{0.33}Zn_{0.33}$-Gly**	23.2	5.73
